# Time to first antenatal care contact and pooled prevalence among reproductive-age women in East Africa: Log-logistic shared frailty model

**DOI:** 10.1371/journal.pone.0325002

**Published:** 2025-06-12

**Authors:** Simachew Getaneh Endalamew, Fetlework Gubena Arage, Asefa Adimasu Taddese, Dejen Kahsay Asgedom, Bewuketu Terefe, Solomon Keflie Assefa

**Affiliations:** 1 Department of Veterinary Epidemiology and Public Health, School of Veterinary Medicine, Bahir Dar University, Bahir Dar, Ethiopia; 2 Department of Biostatistics and Epidemiology, Institute of Public Health, College of Medicine and Health Science, University of Gondar, Gondar, Ethiopia; 3 Department of Public Health, College of Medicine and Health Science, Samara University, Samara, Ethiopia; 4 Department of Community Health Nursing, School of Nursing, College of Medicine and Health Science, University of Gondar, Gondar, Ethiopia; Kwame Nkrumah University of Science and Technology College of Health Sciences, GHANA

## Abstract

**Background:**

Antenatal care (ANC), the provision of prenatal healthcare by skilled medical practitioners, is essential for ensuring the well-being of pregnant women and their fetuses. It reduces maternal and child morbidity and mortality. However, there is a lack of comprehensive, region-wide analyses of ANC initiation, particularly across diverse East African countries. Therefore, this study aimed to determine the time to first antenatal care and its associated factors in East African countries.

**Methods:**

Demographic and Health Survey (DHS) data from 2012–2022 consisting of 12 countries were extracted. A total of 93,213 weighted reproductive-age women (15–49 years) were included in this study. A Kaplan–Meier survivor curve was generated to estimate the time of the first antenatal care contact. A log-rank test was used to compare the difference in survival curves. The log-logistic gamma shared frailty model was selected based on the reduced Akaike and Bayesian Information Criteria, and Cox-Snell residual plot. The shared frailty model was utilized to capture the correlation of outcomes within clusters (countries), as individuals within the same country may experience similar risks.

**Results:**

The pooled prevalence of women with a minimum of 4 ANC contacts in East African countries was 57.7% (95% CI: (49.9–65.1%). The variability in effect sizes of ANC utilization across included countries is estimated at $\tau^{2}=$ 0.2032 [95% CI: 0.1111–0.6611]. The overall median time to the first antenatal care contact was 4 months. The log-logistic shared frailty model showed that place of residence [ϕ = 1.014, 95% CI: (1.006, 1.021)], maternal age [ϕ = 0.978, 95% CI: (0.970, 0.980)], women’s education level [ϕ = 0.964, 95% CI: (0.952, 0.971)], marital status [ϕ = 0.970, 95% CI: (0.963, 0.978)], wealth index [ϕ = 0.990, 95% CI: (0.982, 0.997)], healthcare distance [ϕ = 0.975, 95% CI: (0.969, 0.980)], and parity [ϕ = 1.111, 95% CI: (1.093, 1.129)] were significant determinants of time at first antenatal care visit.

**Conclusion:**

Women in East Africa initiated their first ANC visit later than the optimal period recommended by the World Health Organization (WHO). The positive correlation between early ANC initiation and access to media, healthcare access, and educational attainment may be utilized to promote increased early engagement in ANC services. Thus, governments and other responsible bodies should strive to implement programs to enhance access to healthcare and education, particularly for women living in rural areas, to improve the early initiation of antenatal care visits.

## Introduction

Antenatal care, the provision of prenatal healthcare by skilled medical practitioners, is essential for ensuring the well-being of pregnant women and their fetuses [1]. The timing of the initial ANC visit influences the women’s and children’s health later in life. Early initiation of ANC allows for more frequent maternal and fetal assessments, providing healthcare professionals with greater opportunities to monitor and evaluate fetal and maternal health [2]. Furthermore, it offers healthcare providers a platform to deliver relevant and up-to-date information on prenatal care, proper nutrition, lifestyle adjustments, and potential risks, empowering pregnant women to make informed decisions and take a proactive role in their maternal health [2–5]. The WHO reports that almost 95% of all maternal deaths occurred in low- and middle-income countries (LMICs) in 2020, with many of these deaths being preventable through proper healthcare interventions, including timely ANC contact [6].

Delayed initiation of ANC contact is the main reason for higher maternal and infant mortality due to pregnancy complications in LMICs [7]. It raises the risk of adverse pregnancy outcomes, including perinatal death, stillbirth, early neonatal mortality, maternal death, and complications during pregnancy and childbirth [8–10]. About 75% of maternal deaths occur during the pregnancy period, primarily due to severe bleeding, infections (usually after childbirth), high blood pressure during pregnancy (pre-eclampsia and eclampsia), complications from delivery, and unsafe abortion, which can be either prevented or treated during the ANC period [1].

East Africa has the highest maternal mortality rate among developing regions, with 351 deaths per 100,000 live births [11]. Limited access to quality ANC is a significant contributor to maternal mortality [12]. To address this, the Ending Preventable Maternal Mortality (EPMM) initiative aims to reduce the global maternal mortality ratio (MMR) to below 70 per 100,000 live births by 2030. Countries are encouraged to reduce their MMR by at least two-thirds from 2010 levels, ensuring no country exceeds 140 maternal deaths per 100,000 live births [13]. Success hinges on pregnant women adhering to the WHO-recommended ANC schedule, as timely initiation is key to early detection and prevention of maternal and infant complications [14]. Despite being crucial for reducing maternal and infant mortality, timely ANC initiation remains a challenging issue for many pregnant women, particularly in East Africa [15].

According to the WHO, the optimal time to initiate ANC is during the first trimester (up to 12 weeks of gestation) [1]. Globally, the timing of ANC initiation varies significantly, with marked disparities between regions. The median timeframe for the initial antenatal care appointments among pregnant women in Ethiopia [16] and India [17] was four months. In Southern Western and Southern Nigeria, the mean gestational age at the time of booking was approximately 20.3 weeks, with a standard deviation of 6.2 weeks [18] and 18.3 weeks [19] respectively, while in northern Uganda the mean gestational age at booking was around 22.6 weeks, with a standard deviation of 5.7 weeks [20].

Previous studies have identified various factors influencing the timing of the first ANC contact. These determinants can be grouped into three key categories: socio-demographic factors (maternal age, marital status, place of residence, and head of household characteristics), obstetric factors (parity and Pregnancy intention (wanted vs. unwanted child status)), and socio-economic factors (wealth status, distance to healthcare facilities, media exposure, maternal and husband’s education, and the mother’s employment status) [21–23].

The provision of quality maternal health care in East Africa is challenged by a combination of structural, economic, and sociocultural challenges [23–25]. Geographic and infrastructural barriers, including long distances to health facilities, inadequate road networks, and unreliable transportation systems, frequently restrict timely access to care, particularly in rural areas [26]. Economic constraints further exacerbate this issue, as the high financial burden associated with healthcare services and transportation costs significantly discourage women from utilizing antenatal and delivery services [27]. Sociocultural norms and beliefs also play a pivotal role in shaping health-seeking behaviors, with some cultural practices discouraging the use of institutional maternal health services [28,29].

Moreover, the healthcare infrastructure in the region remains critically under-resourced [24,30]. Many health facilities lack the requisite human resources, medical supplies, and essential emergency obstetric services to address pregnant mothers’ needs. For instance, in 2016, only 16% of Kenyan health facilities were equipped to provide comprehensive emergency obstetric care, reflecting systemic inadequacies in service delivery [31]. These infrastructural limitations are exacerbated by the “three delays” model of maternal mortality: delays in recognizing the need for care (seeking care), delays in reaching healthcare facilities (reaching care), and delays in receiving appropriate care (receiving care) upon arrival [32].

There is a lack of comprehensive, region-wide analyses of ANC initiation, particularly across diverse East African countries. Existing research often focuses on individual country-level data, limiting the ability to compare trends and identify broader regional patterns. This gap is evident in East African countries, where regional variations in ANC initiation are underexplored, limiting the development of targeted interventions and improvements in maternal healthcare through regional collaboration. Moreover, many of these studies have been confined to specific districts, limiting their representativeness at the national level [33–35]. This study aims to determine the time to first ANC contact and identify factors associated with early ANC initiation among women in East African countries. Furthermore, it seeks to identify regional variations in ANC utilization by pooling their effect sizes, providing insights to inform targeted interventions and improve maternal healthcare in the region.

## Methods and materials

### Study setting, study population, and data source

The study was conducted in East African nations and employed a community-based cross-sectional survey design. The study incorporates DHS data from 2012–2022 from 12 countries. The specific East African countries included in this study were Burundi, Ethiopia, Kenya, Comoros, Madagascar, Malawi, Mozambique, Rwanda, Tanzania, Uganda, Zambia, and Zimbabwe. The most recent standard DHS reports available for these countries were obtained and used as the primary data source for the study. All reproductive-aged women (15–49 years) in these countries were the source populations, whereas all reproductive-aged women (15–49 years) in the selected enumeration area (EAs) were the study population.

The DHS program was collaborated with various national institutions to ensure that all surveys adhere to the ethical standards and guidelines set by the country’s legislation. Stringent protocols were followed in DHS to protect the rights and privacy of participants. These protocols were included obtaining informed consent, maintaining confidentiality, and ensuring the anonymity of collected data. The surveys were designed to uphold high ethical standards and prioritize the well-being and safety of participants. The DHS program is expected to gain wide recognition and endorsement from international organizations, including the United Nations and the World Health Organization, as a valuable source of data for evidence-based policies and programs. No additional microdata beyond those utilized in this study was manipulated or applied. There was no involvement of patients or the general public in the study.

### Inclusion and exclusion criteria

The study targeted all pregnant women aged 15–49 years, including those with recorded gestational age, either at their initial ANC visit or for those who did not attend ANC at the time of delivery or pregnancy termination. This inclusion ensured the comprehensive coverage of women with known gestational ages, regardless of ANC attendance. However, women whose gestational age was unrecorded or undetermined at their initial ANC visit were excluded to maintain data accuracy and reliability.

### Data extraction, quality control and Sampling methods

Following an online request explaining the study’s purpose and obtaining authorization, data for the specified countries was extracted using STATA (version 17) from the official DHS program database (https://dhsprogram.com). The analysis focused on the individual’s record (IR file) dataset, extracting dependent and independent variables. This program has been a crucial source of data on reproductive health issues in low and middle-income countries, providing information on topics such as marriage, fertility, fertility preferences, and contraception [36].

The DHS dataset was collected using structured and pre-tested comprehensive standard questionnaires. Data quality was ensured through the implementation of training for data collectors, supervisors, and field editors; conducting ongoing supervision; employing standardized and translated questionnaires in international, national, and country-specific local languages; and engaging data processing specialists for data entry and management. Throughout the process, measures were taken to address systematic bias. More detailed guidance on the data collection process can be found in DHS documentation [37].

**Fig 1:**
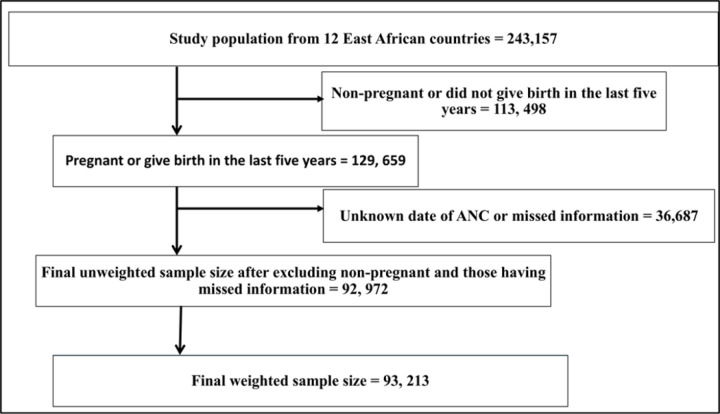
Illustration of sampling procedure from 12 East African Countries using their recent DHS.

The DHS program employed two-stage stratified sampling techniques to select households for the survey. EAs were randomly selected in the first stage, while households were randomly selected in the second stage. All women between the ages of 15 and 49, who were either permanent residents or overnight visitors in the selected households, were eligible to participate in the study. A total of 243,157 women aged 15–49 years were interviewed for the survey with a response rate of 95%. A total of 93,213 weighted reproductive-age women (15–49 years) from 12 East African countries were included in this study (Fig 1).

### Study variables

In this study, the time of the initial ANC contact, measured in months, was the dependent variable. The aim was to investigate the factors that influence the time of initiation of ANC contact. Several factors were considered in the analysis to identify determinants of time to ANC contact. Clusters, Enumeration areas, or clusters were used as clustering variables in all frailty models. This approach accounts for the potential correlation or similarities within communities.

The independent factors included in the analysis are residence, maternal age, marital status, maternal and husband education level, parity (number of previous pregnancies), wealth index, sex of the household head, health insurance coverage, media exposure (specifically, listening to radio, watching television, and reading magazines or newspapers), distance to the health facility, husband’s education level, work status, pregnancy intention, and the number of children.

### Definitions for some operational terms

In the context of this study, the following definitions and statistical models were utilized

#### Event.

This refers to the occurrence of at least one ANC contact during the pregnancy period of women.

#### Censored.

This refers to cases where the pregnant mother did not receive any ANC visits during her entire pregnancy. In addition, if the gestational age or length of pregnancy is known due to birth or pregnancy termination, it is noted as censored data.

#### Survival time.

In this study, survival time is defined as the number of months it takes from the start of pregnancy to the first ANC booking, representing the gestational age at the time of the initial ANC contact.

### Ethical consideration

This study used a publicly available secondary survey dataset from the Measure DHS Program. Therefore, ethical approval and participant consent were not required. Written permission letters were secured from the DHS program data archivists to download and use data for this study. The DHS data were kept confidential, and any identifying information was removed. The data were only used for this authorized research project and would not be shared with researchers.

### Data management and analysis

Before conducting any statistical analysis, appropriate data management procedures, including missing data management, data weighting, and recoding, were implemented using R software. Missing data were initially examined utilizing graphical methods or visual plots to identify patterns. For missing patterns value <5%, Little’s MCAR test was employed to assess whether the data were missing completely at random, which indicated that they were missing at random (MAR) (p < 0.05). Consequently, Multivariate Imputation by Chained Equations (MICE) with logistic regression was utilized to generate 10 imputed datasets using R software. To ensure the robustness of the findings, a sensitivity analysis was conducted to compare results from the imputed datasets with those from a complete case analysis. The data were weighted using sampling weights to ensure representativeness and obtain reliable estimates before conducting any statistical analysis. The weighted results of the analysis were reported in this study.

Statistical analysis was then conducted using R statistical software version 4.4.2 for the imputed dataset. Descriptive metrics such as percentages, graphs, and frequency tables were used to summarize the data and characterize the study population. To estimate the time to the first ANC contact, the nonparametric Kaplan-Meier (K-M) test was utilized. This non-parametric method allows for the estimation of survival probabilities over time. The log-rank test was applied to assess differences in survival times across categorical variables and the desired outcome. For fitting survival data and incorporating factors, both the Cox Proportional Hazard Model and the Accelerated Failure Time (AFT) model were considered. Additionally, shared frailty models, which account for unobserved random effects affecting the survival function, were employed. In the analysis, variables found to be significant (with a p-value of less than 0.25) in the uni-variable analysis were included in the multivariable analysis. The selection of the optimal model was based on criteria such as the Akaike Information Criterion (AIC), Bayesian Information Criterion (BIC) and log-likelihood.

### The Cox proportional hazard model

Cox regression model or the Cox proportional hazards model is a semi-parametric mode1, in which no assumptions are made about the actual form of the baseline hazard function $h_{o}(t)$, introduced by Cox in 1972 [38].

The general proportional hazards model is given by:

$h_{i}(t)=\exp^{\left(\beta 1x1+\beta 2x2+\ldots +\beta pxpi\right)}h_{o}(t)$*,* or in matrix form, it can be written as:

$h_{i}(t)=\exp^{\beta xi}\;h_{o}(t)$*,* where $\beta =\left(\beta_{1},\;\beta_{2},\;\beta_{3}\ldots .\beta_{p}\;\right)$ is the vector of coefficients of the p explanatory variables in the model, $x_{i}$ is the vector of values of the explanatory variables for the $i^{th}\;$individual, whose components are${\;x}_{1i,}$ ${\;x}_{2i}$*,* ${\;x}_{3i}$*,...,* ${\;x}_{pi}$*,* and $h_{o}(t)$=baseline hazard function.

When the Cox regression model is used in the analysis of survival data, there is no need to assume a particular form of probability distribution for the survival times. As a result, the hazard function is not restricted to a specific functional form, and the model has flexibility and widespread applicability [39,40].

### The general accelerated failure time model

When the proportional hazards assumption doesn’t hold, the accelerated failure time model becomes a valuable alternative for studying time-to-event data. This model assumes that individual characteristics affect the time scale multiplicatively, influencing how quickly someone moves along the time axis. Essentially, it allows one to interpret the model in terms of disease progression speed, providing an intuitively appealing way to understand how different factors impact the rate at which an individual experiences events related to the condition [39,41].

As in the proportional hazards model, the baseline hazard function, $h_{o}(t)$, is the hazard of the outcome of interest at the time t for an individual for whom the values of the p explanatory variables are all equal to zero. According to the general accelerated failure time model, the hazard function of the $i^{th}\;$ individual at time t, $h_{i}(t)$is then such that:


$$h_{i}(t)=e^{\eta_{i}}h_{o}\left(\frac{t}{e^{\eta_{i}}}\right)$$

W*here,* $\eta_{i}=a_{1}x_{ij}+a_{2}x_{ij}+\ldots +a_{p}x_{pi}$ is the linear component of the model, in which $x_{ij}$ is the value of the$\;j^{th}\;$ explanatory variable, $\;x_{j\;;}\;j=1,\;2\ldots .p$, for the $i^{th}\;$ individual, i=1, 2….n

The acceleration factor (*ϕ)* and the corresponding survivor function for the $i^{th}$ individual is given by the following equations [39], respectively.


$$\;\;\;\;\phi \;=\;\exp^{ai}\;\;\;$$

$Si(t)=S_{o}\left\{\frac{t}{\exp^{\eta i}}\right\}$, where, ${\;s}_{o\;}(t$is the baseline survivor function

### Multivariable Shared frailty models

Frailty models are the survival data analog to regression models, which account for heterogeneity and random effects. A frailty is a latent multiplicative effect on the hazard function and is assumed to have unit mean and variance *θ*, which is estimated along with the other model parameters [42]. A frailty model is a heterogeneity model where the frailties are assumed to be individual or cluster-specific. In general, it is a random effects model where the frailties are common (or shared) among groups of individuals or spells and are randomly distributed across groups [43–45].

Frailty serves as a useful tool to incorporate random effects within a model, enabling the consideration of associations and unobserved differences among individuals or groups. At its core, frailty represents an unseen or unmeasured random element that alters the hazard function of either an individual or a cluster of individuals in a way that multiplies or modifies it. This latent factor essentially accounts for variations among individuals or groups that might affect how likely they are to experience an event of interest, such as survival or failure, even when these differences aren’t directly measured or observed in the data [46].

To formulate a shared frailty model for survival data, suppose that there are *g* groups of individuals with$\;n_{i\;;}$ individuals in the $i^{th}\;$ group i=1, 2….g. For the proportional hazards model, the hazard of death at time *t* for the $j^{th}\;$ individual, $j=1,\;2\ldots .n_{i}$, in the ${\;i}^{th}\;$group is then given by:


$$h_{ij}(t)=z_{i\;}\exp^{\beta x_{ij\;}}\;h_{o}(t)$$

where $x_{ij\;}i$s a vector of values of *p* explanatory variables for the $j^{th}\;$ individual in the$i^{th}$ group; ***β*** is the vector of their coefficients; $h_{o}(t)$ is the baseline hazard function; and the $z_{i\;\;}$ are frailty effects or random effects that are common for all $n_{i}$ individuals within the $i^{th}$ group [39].

The hazard function in the Equation can also be written in the form:


$$h_{ij}\;=\exp^{(\beta x_{ij}\;+u_{i})\;}h_{o}(t)$$

Where,

$u_{i\;}=\log\left(z_{i}\right)$, and are assumed to be realizations of *g* random variables $u_{1\;}$*,* $u_{2}$*…,* $u_{g\;}$. The distribution assumed for $u_{i\;}$ is taken to have zero mean, and the normal distribution is a common choice.

When the frailty or random effect (Z) is greater than one (Z > 1), it indicates an increased risk and when frailty is below one ((Z < 1) it shows a decreased risk of hazard for the cluster. But, if the proportional hazards assumption is not satisfied, the accelerated failure time frailty model can be used [43].

### Accelerated failure time frailty model

The concept of “accelerated failure time-shared frailty models” refers to a statistical methodology used in survival analysis to model the time until an event occurs while considering unobserved heterogeneity among individuals. These models combine the principles of AFT models and shared frailty models. The model that incorporates this unobserved heterogeneity takes the form of an AFT model with shared frailty is given by [43,47]:


$$\log T_{ij}=\mu +x_{ij\;}^{\prime }\alpha +z_{i}+\sigma \varepsilon_{ij}$$

With $T_{ij}\;$the event time for the subject j from cluster i, μ the intercept, $x_{ij\;}^{\prime }$ the vector of covariates for the subject j from clusteri, α the vector containing the covariate effects, σ the scale parameter, $\varepsilon_{ij}$ the random error term for the subject j from cluster i, and finally the$\;z_{i}$’s are the cluster-specific random effects that are assumed to be identically and independently distributed random variables with density function$f\left(z_{i}\right)$. The random error term is assumed to have a fully specified distribution. Different assumptions have been proposed leading to event time distributions other than the Weibull (e.g., gamma, inverse Gaussian, lognormal, and log-logistic) [47].

In frailty models for survival analysis, three fundamental assumptions govern the behavior of the frailty term. First, frailty independence posits that the frailty term ($z_{i}$) is independent of the covariates ($x_{ij\;}^{\prime }$) and follows a cumulative distribution function (F), characterized by an unknown parameter (θ) [48]. Second, to ensure identifiability, the frailty distribution (F) is selected such that the model remains identifiable, typically by employing a distribution with a fixed expectation (mean), often set to 1. This ensures that the effects of frailty can be distinguished from other model components. Third, the assumption of noninformative censoring stipulates that, given the covariates ($x_{ij\;}^{\prime }$) and frailty ($z_{i}$), censoring is independent and noninformative with respect to the frailty, regression parameters (β), and baseline hazard ($h_{o}(t)$) [49].

In addition to the widely used Cox proportional hazards model [38], AFT models offer two main advantages. First, AFT models provide a simple interpretation of regression parameters, as the log-linear formulation allows the regression coefficients to represent the rate at which failure times are “accelerated” or “decelerated” by covariates, making the relationship between covariates and time-to-event outcomes easier to understand [50]. Second, AFT models are robust to omitted covariates. When relevant covariates are missing, the estimates of regression parameters for the AFT model are less biased compared to the Cox model, which is more sensitive to omitted variables [51].

For this study, the frailty parameter in a shared frailty model represents unobserved heterogeneity at the country level, accounting for the correlation between individuals within the same country who may share common, unmeasured risk factors that influence the time to ANC initiation. It introduces a random effect that captures country-specific variability, addressing the dependency structure among individuals within each country. By modeling this shared risk, the frailty parameter ensures that the analysis reflects both individual- and country-level influences on the time to ANC initiation [52].

In empirical applications, the frailty parameter adjusts for clustering effects, ensuring that the estimates of individual-level covariate effects on the time to ANC initiation are not biased by the unmeasured characteristics of countries. It facilitates more precise estimation by isolating the influence of country-specific factors, such as healthcare access, cultural practices, and policy differences, which could affect the time to ANC initiation but are not directly measured [53].

## Results

### Socio-demographic, economic, and obstetrical characteristics of respondents

The distribution of pregnant women in this study showed variation across East African countries. Most participants, 13,448 (14.50%), were from Malawi. Conversely, Comoros had the lowest number of participants, 2,016 (2.21%). Tanzania and Rwanda followed with 5,837 (6.26%) women and 6,302 (6.76%) women, respectively (Table 1).

**Table 1:** Description of surveys and sample size characteristics of East African Countries.

Countries	Survey Year	Sample size(n)		Weighted percentage (%)
		Un- weighted	Weighted	
Burundi	2016/17	8,660	8,941	9.59%
Ethiopia	2016	7,193	7,590	8.14%
Kenya	2022	10,303	9,392	10.08%
Comoros	2012	2,016	2,065	2.21%
Madagascar	2021	9,315	9,232	9.90%
Malawi	2015/16	13,448	13,515	14.50%
Mozambique	2015	7,623	7,874	8.45%
Rwanda	2019/20	6,167	6,302	6.76%
Tanzania	2022	5,779	5,837	6.26%
Uganda	2016	10,263	10,152	10.89%
Zambia	2018	10,263	7,325	7.86%
Zimbabwe	2015	4,833	4,988	5.35%

A total of 93,213 women who became pregnant in East African countries during the five-year survey period were included. Among them, 86,736 (93.05%) received their first ANC visit, representing the events in the study, whereas 6,477 (6.95%) did not receive their first ANC visit and were considered right-censored. A significant proportion of 20,657 (22.16%) included in the study had no formal education. Among these women, 17,058 (18.30%) initiated ANC contact (events) during their pregnancies. Regarding the educational status of the husbands, 19,107 (20.50%) had no formal education. A large proportion, 52,809 (44.44%), had attended primary education (Table 2).

**Table 2:** Socio-demographic and obstetric characteristics of pregnant women in East African Countries, from their recent DHS (n = 93,213 (weighted).

Covariates	Categories	Weighted Frequency and Percentage	
		Censored number (%)	Event number (%)
Residence	Urban	582 (0.62%)	20723 (22.23%)
	Rural	5894 (6.32%)	66013 (70.82%)
Household head sex	Male	5053 (5.42%)	65272 (70.03%)
	Female	1424 (1.53%)	21463 (23.03%)
Maternal age	15-24	1726 (1.85%)	27953 (29.99%)
	25-34	2833 (3.04%)	39238 (42.09%)
	35-49	1918 (2.06%)	19545 (20.97%)
Maternal education	Not educated	3599 (3.86%)	17058 (18.30%)
	Primary	2365 (2.54%)	42908 (46.03%)
	Higher	513 (0.55%)	26770 (28.72%)
Wealth index	Poor	3916 (4.20%)	35926 (38.54%)
	Middle	1222 (1.31%)	16776 (18.00%)
	Rich	1339 (1.44%)	34034 (36.51%)
Marital status	Unmarried	969 (1.04%)	15122 (16.22%)
	Married	5508 (5.91%)	71614 (76.83%)
Total number of children	no child	98 (0.11%)	748 (0.80%)
	1-5	4810 (5.16%)	74678 (80.12%)
	6-15	1568 (1.68%)	11310 (12.13%)
Pregnancy intention	wanted then	4488 (4.81%)	57397 (61.58%)
	wanted later	1236 (1.33%)	22047 (23.65%)
	wanted no more	753 (0.81%)	7292 (7.82%)
Distance	big problem	3947 (4.23%)	33965 (36.44%)
	not a big problem	2530 (2.71%)	52771 (56.61%)
Health insurance	No	6296 (6.75%)	77382 (83.02%)
	Yes	181 (0.19%)	9354 (10.03%)
Current work	No	3753 (4.03%)	32577 (34.95%)
	Yes	2724 (2.92%)	54159 (58.10%)
Husband education	no education	3058 (3.28%)	16049 (17.22%)
	primary education	2581 (2.77%)	38846 (41.67%)
	higher education	838 (0.90%)	31841 (34.16%)
Parity	Primiparous	967 (1.04%)	20569 (22.07%)
	Multipara	3447 (3.70%)	51348 (55.09%)
	Grand-para	2062 (2.21%)	14819 (15.90%)
Media exposure	no media exposure	4098 (4.40%)	30123 (32.32%)
	have media exposure	2378 (2.55%)	56613 (60.74%)

Among those who had problems with media access, 4,098 (4.40%) did not have ANC visits and were right-censored. Similarly, among those who reported that media exposure was not a problem, 2,378 (2.55%) individuals did not have ANC visits and were also right-censored. Regarding the wealth index of pregnant women, the largest proportion, comprising 39,842 (42.74%) individuals, fell into the poor category. Additionally, approximately 35,373 (37.95%) women were classified as rich. Within the poor category, 3,916 (4.20%) individuals did not have ANC visits and were considered right-censored. Similarly, among the rich category, 1,339 (1.44%) individuals did not have ANC visits and were right-censored (Table 2).

**Fig 2:**
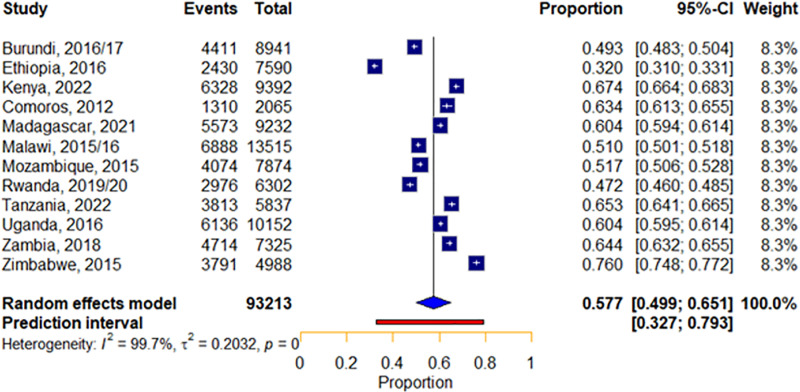
Pooled prevalence of ANC contact in East Africa using the recent DHS 2012 to 2022.

The pooled prevalence of ANC utilization in pregnant women was estimated based on women’s adherence to the World Health Organization’s recommendation of attending a minimum of four ANC contacts during pregnancy. Among 93,213 women included in the study, approximately 52,444 met or exceeded this crucial threshold. The pooled prevalence of women with a minimum of 4 ANC contacts in East African countries was 57.7% (95% CI: (49.9–65.1%). The variability in ANC utilization across countries is estimated at $\tau^{2}=$ 0.2032 (95% CI: 0.1111–0.6611). Furthermore, the results indicated high inter-country variability, as reflected by the inconsistency index ($I^{2}$) of 99.7% (p < 0.01). This substantial heterogeneity suggests that nearly all observed differences in ANC utilization are due to true variability between countries rather than random chance. Zimbabwe recorded the highest ANC utilization at 76%, followed by Kenya at 67.4%. Ethiopia reported the lowest ANC utilization, (32%) (Fig 2).

### Time to first antenatal care booking among pregnant women in East Africa

**Fig 3:**
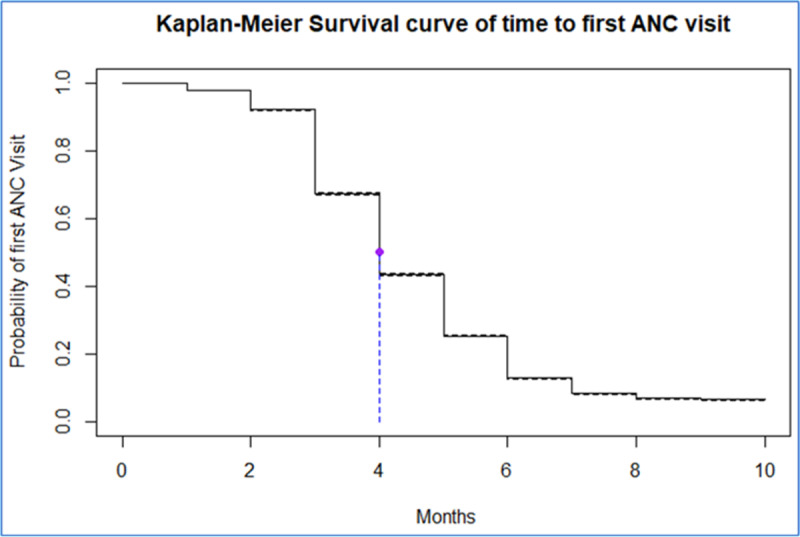
The K-M plots of Survival functions of time to the first ANC contact among pregnant women in East Africa.

The Kaplan-Meier estimate, a non-parametric survival analysis technique, examined the time-to-first ANC contact among pregnant women in East African countries, considering various factors. The probability of starting ANC visits was delayed during the early gestational age. However, as gestational age increases, the probability sharply increases and then gradually declines later. This indicates that pregnant women in East Africa are more likely to initiate ANC contact at the delayed stages of pregnancy. Additionally, the overall median time to the first antenatal care contact was 4 months. This means that, on average, half of the pregnant women in the study population initiated their ANC contacts by the fourth month (Fig 3).

### Comparisons of the different factors in terms of survival time to the first ANC contact

**Fig 4:**
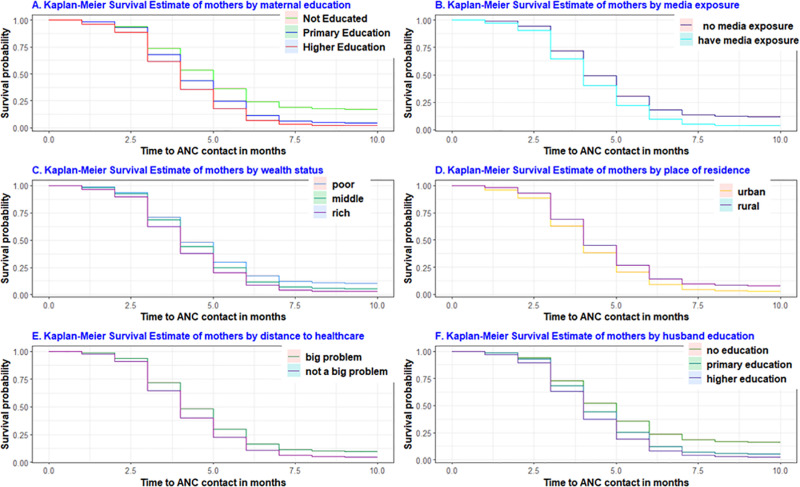
Survival estimate of time to first ANC visit among pregnant women using different factors.

Kaplan-Meier graphs and the log-rank test were conducted to compare differences in survival experiences across categorical variables. In the Kaplan–Meier survival plot, curves positioned below others indicate that the groups represented by the lower curves have a lower survival status compared to those represented by the upper curves (Fig 4).

The log-rank test was also used to assess the statistical significance of these differences. The log-rank test revealed a statistically significant (p < 0.001) disparity in survival outcomes for the place of residence, maternal education, maternal age, wealth index, husband’s education, maternal occupation, marital status, parity, media exposure, and pregnancy intention (Table 3).

**Table 3:** Comparison of survival time, for the first antenatal care visit (in months) among pregnant women in East Africa.

Variable	Log-rank test	P-value	Variable	Log-rank test	P-value
Residence	702.86	< 0.001	Distance	1231.59	< 0.001
Household head sex	11.23	< 0.001	Health insurance	2556.38	< 0.001
Maternal age	168.52	< 0.001	Current work	1100.65	< 0.001
Maternal education	4162.30	< 0.001	Husband education	3188.53	< 0.001
Wealth index	1983.66	< 0.001	Parity	1450.24	< 0.001
Numberof Children	1073.25	< 0.001	Pregnancy intention	183.62	< 0.001
media exposure	2027.54	< 0.001	Marital status	2.48	0.03

### Model adequacy or selection

To evaluate the proportional hazard assumption of the Cox proportional hazards model, diagnostic tests were conducted, including the rho statistic and examination of the scaled Schoenfeld residuals. The rho statistic quantifies the extent of correlation between the model’s residuals and time, with a larger value indicating a stronger association. The proportional hazards assumption was violated, as indicated by significant rho and global tests (p < 0.05) linked to time to first ANC contact, leading to the adoption of AFT models with diverse baseline and frailty distributions (Table 4).

**Table 4:** Assumption of the proportional hazard model.

Covariates	Rho	Chi^2^-test	Degree of freedom	P-value
Residence	0.11	7.53	1	0.0061
Household Head sex	0.01	3.02	1	0.0423
Maternal age	0.06	50.28	2	0.00041
Maternal education	0.22	423.25	2	< 0.001
Wealth status	0.15	96.00	2	< 0.001
Marital status	0.01	22.65	1	0.02467
Numberof Children	0.11	37.80	2	0.02502
Pregnancy intention	0.05	295.44	2	0.01796
Distance	0.12	4.62	1	0.0428
Health insurance	0.17	467.04	1	< 0.001
Current work	0.11	3.88	1	0.0489
Husband education	0.19	373.56	2	< 0.001
Parity	0.13	12.25	2	0.0022
media exposure	0.15	131.38	1	< 0.001
Global Test		1523.94	21	< 0.001

The best model was selected based on the lowest AIC and BIC values, and the highest log-likelihood value. Among the models considered, the log-logistic gamma shared frailty model showed the best performance with the lowest AIC (111,172.1), BIC (111,398.7), and highest log-likelihood (−55,562.07), making it the most suitable for describing factors affecting the time to initiate ANC contacts (Table 5).

**Table 5:** AFT Model comparisons with and without frailty for different baseline distribution.

Model selection Criteria	Models	Frailty distributions	
		No frailty	Gamma shared frailty
AIC	exponential	209027	206945.5
	Weibull	134756.4	126888.3
	log-normal	124225.6	116206.8
	Log-logistic	118776.3	111172.1^a^
BIC	exponential	209234.7	207162.6
	Weibull	134973.5	127114.9
	log-normal	124442.7	116433.3
	log-logistic	118993.4	111398.7^a^
Log-likelihood	exponential	−104491.5	−103449.7
	Weibull	−67355.21	−63420.16
	log-normal	−62089.79	−58079.39
	log-logistic	−59365.16	−55562.07^a^

^a^preferred model.

The rationale for using a shared frailty model is to capture the correlation of outcomes within clusters (countries), as individuals within the same country may experience similar risks due to shared environmental, socio-economic, or healthcare-related factors. By incorporating frailty, the model accounts for this intra-country dependence and provides more accurate estimates of the effects of covariates. It allows for the assumption that individuals within the same cluster may not be independent, thereby improving the robustness of the survival analysis and adjusting for the potential confounding effects that may arise from such clustering.This model offers a flexible parametric approach to survival analysis, enabling the hazard function to exhibit increasing, decreasing, or unimodal patterns over time, which correspond to the observed data. Model diagnostics, including AIC and BIC, confirmed the superior fit of the log-logistic shared frailty model in comparison to alternative models (Table 5). Furthermore, the Cox model’s proportional hazards assumption was potentially violated in preliminary analyses, further substantiating the selection of the log-logistic model for robust and reliable estimates.

**Fig 5:**
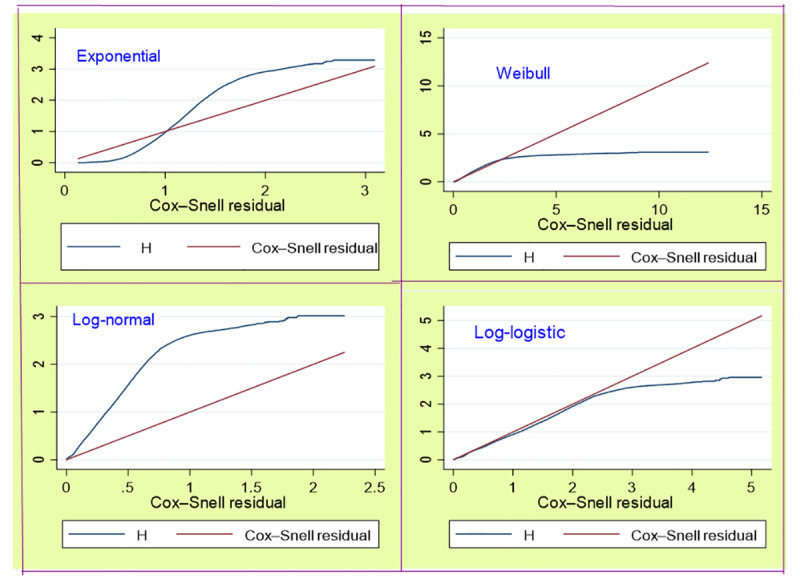
Cox-Snell residual plots against cumulative hazard for exponential, Weibull, log-normal, and log-logistic AFT models.

Finally, the goodness of fit was assessed using Cox-Snell residual plots to examine the alignment between the residual and cumulative hazard functions. When considering exponential, Weibull, and log-normal distributions, the Cox-Snell residuals deviated significantly from the cumulative hazard function. In contrast, the Cox-Snell residuals for the log-logistic baseline distribution exhibited closer proximity to the cumulative hazard function curve (Fig 5). Therefore, this observation substantiates that the log-logistic frailty baseline distribution offers a more favorable fit to the dataset compared to the other distributions examined. Using the AFT model, the univariable analysis identified all variables as significant at a 25% threshold (p < 0.25), and these were subsequently included in the multivariable analysis.

### Log-logistic gamma shared frailty model result

The test problem for the null hypothesis of no unobserved heterogeneity is given by $H0:\;\sigma^{2}=0,\;$versus$\;\;HA:\;\sigma^{2}>0\;\;$where$\;\sigma^{2}$ denotes the frailty variance (unobserved heterogeneity). The frailty in this model is assumed to follow a gamma distribution with mean 1 and variance equal to theta (θ). The frailty variance (θ) = 0. 099 (95% CI: 0. 045, 0. 217) indicates that there is heterogeneity between countries. A likelihood ratio test for the hypothesis θ = 0 indicates a chi-square value of 7606.17 with one degree of freedom resulting in a highly significant p-value of less than 0.001. This implied that the frailty component had a significant contribution to the model.

### Predictors of time to first antenatal care booking

The outcome of the multivariable AFT model using log-logistic distribution as a frailty factor revealed that factors, including residence, marital status, education levels of both the mother and husband, wealth index, media exposure, parity, maternal age, distance to healthcare, possession of health insurance, and current work status, are statistically significant at the 5% level of significance. On the other hand, factors such as the sex of the household head, the total number of children, and whether the pregnancy was desired or not were insignificant to the time it takes for pregnant mothers in East African countries to make their first ANC visit (Table 6).

**Table 6:** Log-logistic gamma shared frailty model results.

Factors	Coef.	SE	Z	ϕ	95% CI of ϕ	P-value
**Residence (Ref = Urban)**						
Rural	0. 014	0.004	3.63	1.014	(1.006, 1.021)	0.000
**Household head sex (Ref = Male)**						
Female	−0.002	0.003	−0.49	0.998	(0.992, 1.005)	0.624
**Maternal age (Ref = 15–24)**						
25-34 35-49	−0. 022	0.004	−10.28	0.978	(0.970, 0.980)	0.000
	−0. 037	0.005	−9.83	0.964	(0.952, 0.971)	0.000
**Maternal education (Ref = Not educated)**						
Primary Higher	−0. 032	0.003	−8.78	0.969	(0.961, 0.975)	0.000
	−0. 039	0.005	−7.77	0.962	(0.952, 0.971)	0.000
**Wealth index (Ref = poor)**						
Middle Rich	−0. 010	0.004	−2.76	0.990	(0.982, 0.997)	0.006
	−0. 025	0.004	−6.46	0.975	(0.969, 0.983)	0.000
**Marital status (Ref = unmarried)**						
Married	−0. 030	0.005	−7.40	0.970	(0.963, 0.978)	0.000
**Total number of children (Ref = no child)**						
1-5 6-15	−0. 017	0. 014	−1.14	0.983	(0.956, 1.012)	0.254
	0. 012	0. 016	0.73	1.012	(0.980, 1.045)	0.463
**Pregnancy intention (Ref = wanted then)**						
wanted later wanted no more	0.073	0.003	22.61	1.076	(1.069, 1.082)	0.000
	0.082	0.005	15.76	1.085	(1.075, 1.097)	0.000
**Distance (Ref = big problem)**						
not a big problem	−0. 025	0.003	−8.67	0.975	(0.969, 0.980)	0.000
**Health insurance (Ref = no)**						
Yes	−0. 119	0.005	−22.47	0.888	(0.879, 0.897)	0.000
**Current work (Ref = no)**						
Yes	−0. 025	0.003	−4.71	0.975	(0.969, 0.981)	0.000
**Husband education (Ref = no education)**						
primary education higher education	−0. 025	0.004	−6.37	0.975	(0.967, 0.983)	0.000
	−0.045	0.005	−9.54	0.956	(0.947, 0.966)	0.000
**Parity (Ref = primiparous)**						
Multipara Grand-para	0. 052	0.004	13.08	1.053	(1.045, 1.062)	0.000
	0. 105	0.008	12.79	1.111	(1.093, 1.129)	0.000
**Media exposure (Ref = no media exposure)**						
have media exposure	−0. 024	0.003	−7.76	0.976	(0.970, 0.982)	0.000

SE= standard error, ϕ = acceleration factor, Ref= reference.

The acceleration factor for time to first ANC visits among mothers who live in rural areas was 1.014 (ϕ = 1.014, 95% CI: 1.006, 1.021) compared to urban dwellers. This means that, after adjusting for other factors and frailty term constant, women residing in rural areas delay ANC contact initiation by a factor of 1.014 compared to urban dwellers. This indicates that the time it takes for rural women to initiate their first ANC visit is longer than that for their urban counterparts.

Women aged 25–34 had an acceleration factor of 0.978 (ϕ = 0.978, 95% CI: 0.970, 0.980), and those over 35 had an acceleration factor of 0.964 (ϕ = 0.964, 95% CI: 0.952, 0.971), using the 15–24 age group as the reference. This indicates that, after adjusting for other factors and maintaining a constant frailty term, women in the 25–34 and 35–45 age groups initiated their first ANC contact 2.2% and 3.6% earlier, respectively, than those in the 15–24 age group. This indicates that both older age groups initiate their first ANC visit significantly earlier than the 15–24 age group.

The acceleration factor for time to first ANC visits among mothers who had secondary or above education levels was 0.962 (ϕ = 0.962, 95% CI: 0.952, 0.971) compared with uneducated mothers. Additionally, the acceleration factor for time to first ANC visits in mothers with primary education was 0.969 (ϕ = 0.969, 95% CI: 0.961, 0.975) compared with the reference group (no education). This means that, after controlling for other variables and maintaining a constant frailty term, mothers with primary education and those with secondary or higher education initiated their first ANC contact 2.2% and 3.6% earlier, respectively, compared to mothers without formal education. This shows that educated women initiate their first ANC visit earlier than uneducated mothers.

Women from the middle and rich wealth index had an acceleration factor of 0.990 (95% CI: 0.982, 0.997) and 0.975 (95% CI: 0.969, 0.983) respectively, compared to the poor groups. This means that, after controlling for other variables and maintaining a constant frailty term, mothers from middle and rich socioeconomic status initiated their first ANC contact 2.7% and 2.5% earlier, respectively, compared to mothers from economically disadvantaged families. This indicates that women who are from rich families initiate their first ANC earlier than the reference category.

Women with multiple parities had an acceleration factor of 1.053 (95% CI: 1.045, 1.062), and women with grand multipara had a higher acceleration factor of 1.111 (95% CI: 1.093, 1.129), compared with nulliparous women as the reference. This implies that, after accounting for other covariates and frailty constant, women with multiple and grand parity take a significantly extended period before initiating their initial ANC visit.

Women with media exposure had an acceleration factor of 0.976 (95% CI: 0.970, 0.982), indicating that, after adjusting for other covariates and accounting for frailty term, they initiated the first ANC visit 2.4% earlier compared to those without regular media exposure. In addition, married women had an acceleration factor of 0.970 (95% CI: 0.963, 0.978), indicating that, after adjusting for other covariates and accounting for frailty term, they initiated the first ANC visit 3% earlier as compared to unmarried women.

The calculated coefficient for the healthcare distance parameter was estimated to be −0. 025, suggesting that proximity to healthcare facilities is a substantial hindrance to access to ANC. The negative sign of the coefficient indicates a decrease in the logarithm of the survival time. In practical terms, this implies a reduction in the expected duration of the time taken to make the first ANC visit when compared to women for whom the distance to healthcare poses a significant obstacle, as denoted by the reference group.

## Discussion

Using recent DHS data, this study assessed the pooled proportion and time to the first ANC visit across twelve East African countries. The median time to initiate the first ANC visit was higher than WHO recommended which is in the first trimester (within 12 weeks of gestation) [1]. The result of the current study is consistent with a study from India [17]. However, this finding is lower than the studies done in Southern Nigeria [19]. The proportion of women with 4 or more ANC visits is considerably lower than the global average of 69% [15], but higher than the studies from India [54] which reported 51.6%. This may be attributed to disparities in healthcare access, socioeconomic conditions, cultural beliefs, and policy effectiveness.

The frailty model used in this investigation accounted for unobserved heterogeneity between countries and captured the within-country correlation in the timing of the first ANC visits. The significant clustering effect (p = 0.000) observed in the log-logistic-gamma shared frailty model demonstrates the presence of substantial variability between countries in terms of healthcare access, cultural norms, and socioeconomic factors, all of which influence the timing of ANC visits. By incorporating this unobserved heterogeneity, the model ensures that the impact of these country-level factors is adequately accounted for, thereby mitigating potential bias in the survival estimates. This emphasizes the importance of considering both individual and contextual factors in shaping maternal health outcomes and suggests that interventions targeting country-specific barriers may play a critical role in improving timely access to ANC services.

Higher maternal and husband education levels, urban residence, living with a husband, increased wealth status, health insurance coverage, occupation (employment), and media accessibility were associated with earlier initiation of ANC visits. In contrast, lower maternal education, lower wealth quintile(s), and higher birth order or parity were associated with delayed initiation of ANC visits.

In line with previous research [55–57], this study showed that younger women tended to delay attendance to antenatal care compared to their older counterparts. This might be attributed to factors such as limited awareness, restricted access to healthcare resources, financial constraints, and societal stigma associated with early pregnancy [58]. Additionally, adolescent girls may face unique challenges in accessing ANC. A qualitative study highlighted that factors including lack of motivation, denial of pregnancy, social norms, policies, and clinic environment can hinder their timely attendance to ANC services [59].

Increased economic status and family educational levels were highly associated with the early initiation of ANC, which coincides with previous literature [54,55,60,61]. This may be attributed to women from the highest wealth quintile having greater financial and social access to healthcare services, which can lead to earlier initiation of the first ANC, as observed in the current analysis [62,63]. In addition, educated women possess a greater understanding of the advantages of utilizing antenatal care services. They recognize the positive impact of ANC in enhancing the health of both mothers and infants by facilitating the early identification of pregnancy-related complications and enabling access to a range of preventive measures and health promotion services [64].

Women residing in rural areas exhibited a delayed onset for their initial ANC visits compared to their urban counterparts. The result was in agreement with the findings from Nepal [65], Bangladesh [21] and Ethiopia [66]. This may be attributed to challenges related to limited healthcare accessibility and increased work demands, particularly since many of them are engaged in agricultural activities that require additional time and effort to balance agricultural work with attending antenatal care appointments [24].

Parity affected the survival times of the first ANC visit, with parous women having longer survival times compared to nulliparous women. Similar findings reported that increasing parity delayed the initiation of antenatal care visits [19,21,67]. A study in Uganda found that 73% of high-parity women sought their first ANC visit after the first trimester, suggesting that prior childbirth experiences might lead these women to perceive early ANC as less essential [68]. Similarly, research in Nepal reported that previous positive pregnancy experiences made pregnant women develop confidence, reducing their motivation to initiate antenatal care early [69]. These findings suggest that increased confidence from prior pregnancies may contribute to delayed ANC attendance among multiparous women.

Marital status played a significant role in the timing of ANC initiation, with married women having shorter survival times as compared to those not living with their husbands. This finding is in line with studies conducted in South Africa and Ethiopia [70,71].The observed trend could be explained by the social support and shared responsibilities within a marital relationship. In line with previous findings [72,73], women with occupations also had shorter survival times as compared with women with no jobs, likely due to better access to healthcare resources, increased awareness of the importance of early prenatal care, and potentially fewer barriers to seeking and utilizing healthcare services among women with occupations.

Furthermore, pregnant women having media exposure began their ANC earlier than those without media exposure, which is consistent with previous studies [74–76]. Research in sub-Saharan Africa [77] reported a strong positive relationship between mass media exposure and maternal healthcare service utilization, suggesting that media campaigns can effectively encourage early ANC attendance. These findings emphasize the importance of media in disseminating information about maternal healthcare, thereby enhancing awareness and prompting pregnant women to seek ANC services earlier in their pregnancies.

### Strengths and limitations of the study

The research utilized nationally representative DHS data, providing a comprehensive perspective on ANC across multiple nations. This extensive coverage enhances the study’s validity and generalizability. Furthermore, the implementation of a frailty model facilitates the examination of time to ANC contact variations, effectively accounting for both observable and unobservable factors.

The limitations of our analysis stem predominantly from the use of secondary data. Surveys were conducted between 2012 and 2022, and all indicators of ANC use and content rely on women’s self-report of events during the pregnancy preceding their most recent live birth, relying on participants’ ability to accurately remember past events. This may introduce maternal recall bias. Finally, the model did not incorporate all possible variables influencing ANC contacts, potentially limiting the comprehensiveness of the analysis and our understanding of ANC contact.

## Conclusion

This study showed that women living in East Africa initiated their first ANC visit later than the optimal period recommended by WHO. These results highlight a regional challenge in early ANC initiation, with increased care-seeking observed as gestational age advances. These findings emphasize the importance of promoting early and timely ANC visits to ensure the well-being of both pregnant women and their infants. Targeted interventions are essential for addressing barriers to early ANC initiation. The governments and other responsible bodies should strive to implement programs to enhance access to healthcare and education to improve early initiation of ANC visits, particularly for women living in rural areas. Therefore, Policy interventions should focus on increasing awareness and knowledge among pregnant women regarding the importance of early ANC utilization, especially within the first 12 weeks of gestation, to detect and address pregnancy-related complications early. Policymakers should consider community-based initiatives to enhance awareness and promote early ANC visits, particularly in rural and underserved areas. The implementation of mobile health services could effectively reach remote populations, providing essential prenatal care and information in situ. Financial incentives such as subsidized transportation and reduced-cost services should be introduced to mitigate economic barriers. Additionally, health education programs and culturally sensitive outreach efforts are crucial for overcoming societal and informational obstacles to early initiation of ANC.

Future research should focus on investigating the factors influencing ANC uptake, such as healthcare infrastructure, cultural norms, and the role of male involvement in maternal health decisions. Longitudinal studies can offer valuable insights into how these factors evolve and impact the timing of ANC visits. Moreover, assessing the efficacy of mobile health interventions, community-based programs, and financial incentives to improve ANC uptake is beneficial. Research examining the impact of policy changes such as universal healthcare access or transportation subsidies could provide critical guidance for developing scalable interventions to improve maternal health outcomes across East Africa and similar regions.

## Supporting information

S1 File (DTA)

S2 File Antenatal care contact dataset. (PDF)

## Abbreviations

95% CI: 95% Confidence Interval
ANC: Antenatal Care
WHO: World Health Organization
DHS: Demographic and Health Surveys
AFT: Accelerated Failure Time
AIC: Akaike Information Criterion
BIC: Bayesian Information Criterion
LMICs: Low- and Middle-Income Countries.
